# Effects of seed provenance, pre-treatment and mass on germinability and seedling growth of *Balanites aegyptiaca* (L.) Delile and *Ricinodendron heudelotii* (Bail.) Pierre in Benin (West Africa)

**DOI:** 10.1016/j.heliyon.2021.e08540

**Published:** 2021-12-03

**Authors:** Guillaume Hounsou-Dindin, Rodrigue Idohou, Marcel T. Donou Hounsode, Aristide Cossi Adomou, Achille Ephrem Assogbadjo, Romain Glèlè Kakaï

**Affiliations:** a Laboratoire de Biomathématiques et d’Estimations Forestières (LABEF), Faculté des Sciences Agronomiques, Université d’Abomey-Calavi, 04 BP 1525, Cotonou, Benin; b Ecole de Gestion et de Production Végétale et Semencière (EGPVS), Université Nationale d’Agriculture, BP 43 Kétou, Benin; c Laboratoire de Botanique et Écologie Végétale (LaBEV), Faculté des Sciences et Techniques (FAST), Université d’Abomey-Calavi, 01 BP 4521, Cotonou, Benin; d Laboratoire d’Ecologie Appliquée (LEA), Faculté des Sciences Agronomiques, Université d’Abomey-Calavi, 01 BP 526, Cotonou, Benin

**Keywords:** Agroforestry species, Domestication, Germination rate, Phytodistrict, Scarification, Wild oil species

## Abstract

*Balanites aegyptiaca* (L.) Delile and *Ricinodendron heudelotii* (Bail.) Pierre are socioeconomically important species in sub-Saharan Africa. This study was conducted to assess the seed germinability and seedling growth of those species based on several treatments and to define proper conservation and domestication strategies in Benin. The seeds were randomly collected in their natural habitats. The experiment was conducted using a split-split plot design and the data was analyzed using the generalized linear mixed and survival models. The heaviest seeds (*B. aegyptiaca* seed mass ≥3 g and *R. heudelotii* ≥ 1.50 g) provided the highest germination rates (73.60 ± 5.19% and 62.50 ± 5.71%, respectively) when seeds were scarified with a hammer. For *B. aegyptiaca* seedlings, the seeds from the phytodistrict of North Borgou scarified with a hammer and the heaviest seeds showed the highest total height (36.43 ± 1.03 cm), basal diameter (2.84 ± 0.03 mm), the greatest number of leaves (32), and ramifications. The heaviest seeds of *R. heudelotii* had also the highest value for total height at the day-28 after sowing (26.73 ± 13.56 cm) until the day-105 (151.97 ± 6.37 cm). The heaviest seeds of *R. heudelotii* from the phytodistrict of Pobe showed the highest basal diameter (12.53 ± 1.47 mm) and the greatest number of leaves (14), with almost no ramification during the trial period. These findings constitute a step forward in upscaling the reproduction of these species for better contribution to economies while serving in restoration plans.

## 1 Introduction

Climate change effects have been identified as the most drastic component affecting biodiversity as well as the ecosystem (Hooper et al., 2005). The wildly available resources from some trees seem not to be sufficient to sustain the growing demand since large numbers of these people are facing chronic food insecurity even in the growing season (Hounsou-Dindin et al., 2018). For the last two decades, shortage in the rainfall has been noticed with a high impact on the ecosystem and has contributed to the extension of several species. Biodiversity needs to be promoted to improve regulation services at the landscape level (soil fertility, water infiltration, etc.) and because, the goods and services provided by the ecosystems are important for local people's livelihoods, their food security, and adaptation to climate changes. In the current context of climate change, the practices are changing to seek ways for mitigation. In Africa, fallows practices have been adopted for several years but this method seems to be inappropriate nowadays due to the high demography pression (Kumar and Nair 2011). Forest plantations initiated in the 1960s using exotic species (*Acacia auriculiformis* A.Cunn. ex Benth., *Gmelina arborea* Roxb., *Tectona grandis* L.f., *Terminalia superba* Engl. & Diels) although not widespread today, are still encountered, but the new trend is towards tree crop species like *Anacardium occidentale* L., *Cocos nucifera* L., *Elaeis guineensis*, Jacq., *Mangifera indica* L.). Other common tree-related land-use types in the country are agroforestry parks, where mature trees of species such as *Adansonia digitata* L.*, Azadirachta indica* A.Juss., *Faidherbia albida* (Delile) A. Chev.), *Irvingia gabonensis* (Aubry-Lecomte ex O'Rorke) Baill., *Parkia biglobosa* (Jacq.) R.Br. ex Benth.*, Vitellaria paradoxa* C. F. Gaertn., are preserved and among which annual crops are cultivated (Assogbadjo et al., 2012; Bayala et al., 2014).

*Balanites aegyptiaca* (L.) Delile and *Ricinodendron heudelotii* (Bail.) Pierre are socio-economically and culturally important tree species for oil production covering the three climatic zones zone in Benin republic. plants that distinctly covered the three climatic zones of Benin: *B. aegyptiaca* is concentrated in the semi-arid zone and *R. heudelotii* (Bail.) covers the sub-humid and humid zones of the country. They are found on a variety of land use across phytodistricts in climatic zones according to their ecological preferences on which the structural characteristics of the stands generally depend (Hounsou-Dindin et al., unpublished data). These trees represent the sources of oil and contribute to income generation for local communities in sub-Saharan Africa. Oils extracted from these plants are potentially rich in essential fatty acids (Omega 3, 6), fat-soluble vitamins (A, D, E, and K), and trace elements essential for human health (Boubacar et al., 2018; Kouame et al., 2012). Unfortunately, the density of these species continues to decrease significantly due to the over-exploitation alongside the climate change effect.

Successful plant domestication strategies in tropical areas involved several steps among which are mainly linked to the species productivity (Boyle et al., 1997), growth, and adaptability (Fredrick et al., 2015). The natural distribution of some trees covers large areas whereas other species have limited natural distribution. Some species show large morphological variation whereas others are more uniform. The phytodistricts reflect a variation in environmental conditions (Adomou et al., 2006) and offer the opportunity to test the relationships between individuals in each area.

According to Padonou et al. (2013), seed size is considered to be an indicator of seed quality alongside the seed mass within a species. Such seed mass variation may affect seedling growth. Previous report showed that large-sized seeds germinate faster than smaller seeds (Das et al., 2016). However, and according to Howell (1981), such a statement is not always verified as in some cases small seeds germinated faster than large seeds, e.g., *Impatiens capensis* Meerb. The germinative aptitude of seeds of *B. aegyptiaca* and *R. heudelotii* and the growth of their seedlings can be influenced by the morphology of the seeds (Padonou et al., 2013) or the fruit of which they come (Idohou et al., 2015).

Several species showed interesting responses to the mechanical/manual pre-treatments of seeds (scarification), chemical (using nitric or sulphuric acid), or thermic treatment (soaking with hot, tepid, pain tap, or cold water) throughout certain times which can go from few minutes to several hours or days (FAO 1992). For many tropical species, special seed pre-treatment is often necessary to improve the germination rate. The pre-treatments do not germinate the seeds but make them capable of germinating later when all the required conditions are met (FAO 1992). As reported by Kouyaté et al. (2015) and Kouame et al. (2012), *B. aegyptiaca* and *R. heudelotii* are recalcitrant seeds henceforth need pre-treatment to improve and increase the chances of germination. These pre-treatments through mechanical, chemical, or physiological treatment may have helped to break the dormancy (FAO 1992). In this study, only the mechanical/manual and thermic pre-treatments of seeds were tested.

This study was undertaken to compare the rates of germination and seedling growth, based on the origin of the seed, seed mass, and pre-treatments. Specifically, the research aims to: (i) describe the variability in the seed mass of *B. aegyptiaca* and *R. heudelotii* in the study zones, (ii) assess the effect of seed origin, seed mass, and seed pre-treatments on the germination rate of the target species, and (iii) assess the effect of the seed origin, seed mass, and seed pre-treatments on seedling growth for their large-scale production.

## 2 Methodology

### 2.1 Study area

**Figure 1:**
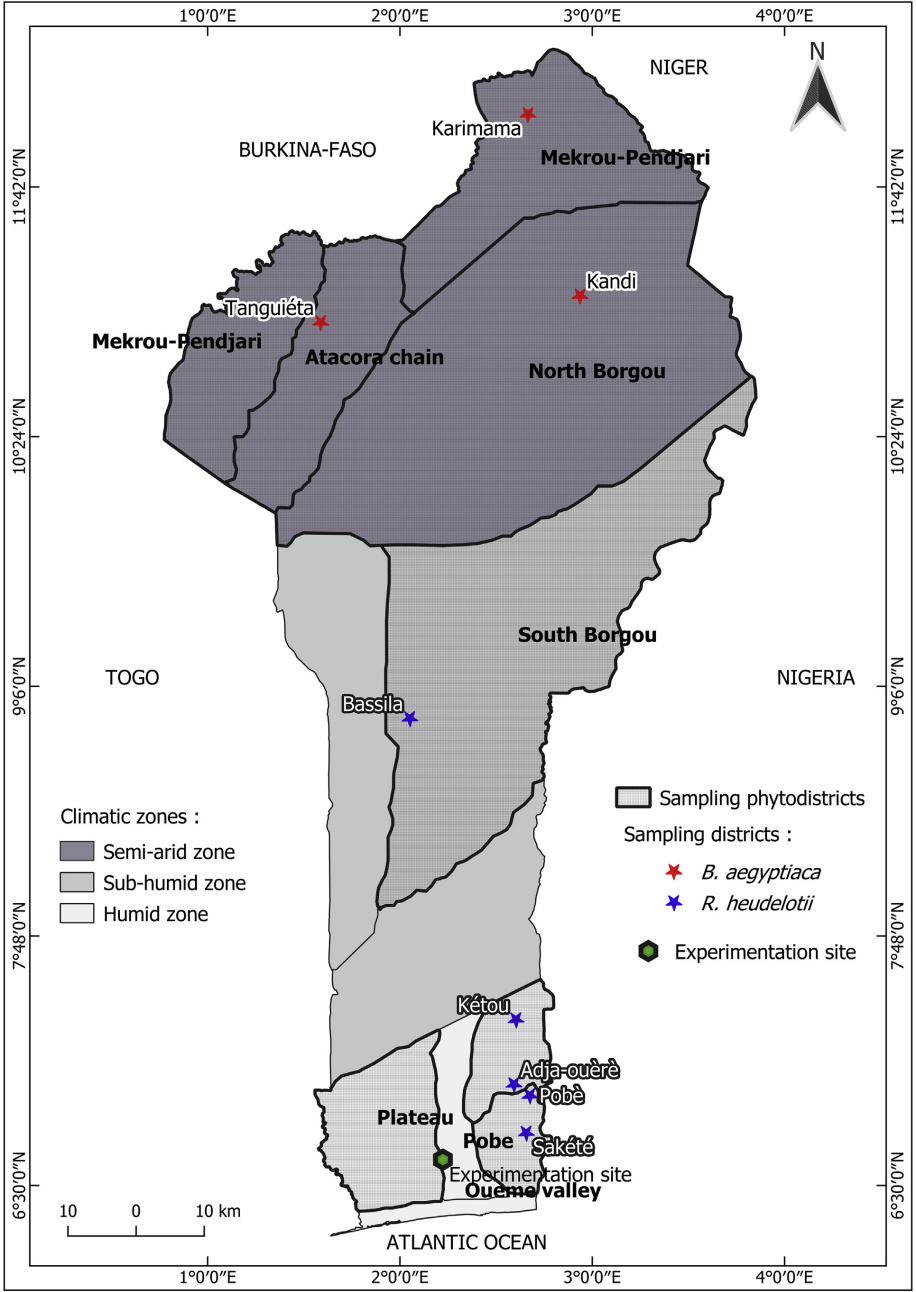
Benin map showing the geographical location of the localities surveyed for seed collections and the experimentation site.

The trial was carried out on the experimentation site of the Laboratory of Biomathematics and Forest Estimations (LABEF), located in the subdistrict of Sékou (district of Allada), in the phytodistrict of Oueme valley. The climate is of the subequatorial type with two (02) rainy seasons, a large season, from March to June and a small season, from September to November, and two (02) dry seasons, from July to September and from November to March. The mean annual rainfall is 1100 mm with ferrallitic soils (Adomou et al., 2006). The seeds were collected in the phytodistricts of the species occurrence in Benin. Three phytodistricts were prospected for each species: Atacora chain, Mekrou-Pendjari, and North Borgou phytodistricts in the semi-arid zone for *B. aegyptiaca*; South Borgou phytodistrict in the sub-humid zone, and Plateau and Pobe phytodistricts in the humid zone for *R. heudelotii* (Figure 1). These phytodistricts were those in which *B. aegyptiaca* and *R. heudelotii* are present and abundant (Hounsou-Dindin et al., unpublished data).

### 2.2 Sampling and fruits’ collection

Each phytodistrict (Atacora chain, Mekrou-Pendjari and North Borgou phytodistricts for *B. aegyptiaca* and South Borgou, Plateau and Pobe phytodistricts for *R. heudelotii*) was prospected for seed collection (Figure 1). A total of thirty (30) adult trees at the stage of optimal morphological and physiological maturity (in the fruiting period) were randomly selected in each phytodistrict for *B. aegyptiaca.* For *R. heudelotii*, ten (10) trees were randomly selected per phytodistrict. For each species, a distance of at least 100 m was considered between sampling trees to minimize the possibility of collecting seeds from the same and closest trees. A total of 30 ripe and unaffected fruits was randomly collected per tree and stored in dry bags per tree. The weight of the collected 30 sampled fruits per tree were measured using a precision balance (sensitivity 0.01 g). Finally, nine plastic pots were used to soak the seeds for the three seed mass classes of each phytodistrict. Four seeds were selected per seed mass class, with 12 per pre-treatment, 24 per phytodistrict, and 72 per block. A total of 216 seeds were used for the 54 experimental units for each species.

### 2.3 Experimental design and data collection

The seeds of both species were planted after the dormancy in the seed have identified to be broken. As such, two recommended (environmentally friendly presenting less or no health risks and technically and financially accessible to all) pre-treatments were tested. Seeds of each species were soaked in tap water for 72 h (T1) and scarified with a hammer (T2). This manual scarification (T2) consists of physically opening (slightly damaged) the seed coat to allow moisture and air in. Both pre-treatments were identified as adequate with high germination rates: 50–90% for *B. aegyptiaca* (Boubacar et al., 2018; Kouyaté et al., 2015) and 63–88% for *R. heudelotii* (Djeugap et al., 2013; Kouame et al., 2012). Viable seeds (i.e., able to germinate when conditions are right, provided that any dormancy has been broken) were selected from a viability test following the method of (Assogbadjo et al., 2010). This test involves submerging the seeds in plain water for 24 h and removing any that float on the surface. The separation of viable seeds and their pre-treatment ensure a high germination rate.

The seeds were sown in black polyethylene bags (12 × 18 cm) filled with substrate from the test site (Sékou) with one seed per pot at a depth of 1–1.5 cm from the surface. The pots were aligned in the nursery using a split-split plot with three replicates. The main factor was the phytodistrict and the secondary factors were the pre-treatments and seed mass classes. The factors were randomized per unit. A total of 216 pots were used per species for the alignment of the 18 treatments (03 phytodistricts × 02 pre-treatments × 03 seed mass classes) considered in the trial (Table 1), at the rate of 04 pots per treatment with the three replicates. The pots were watered for 3 days before sowing.

**Table 1:** Factors tested in the frame of the experiment and their modalities.

Factors	Modalities	
	*B. aegyptiaca*	*R. heudelotii*
Phytodistrict	- Atacora chain - Mekrou-Pendjari - North Borgou	- Plateau - Pobe - South Borgou
Seed mass class (P)	- P1 - P2 - P3	
Pre-treatment (T)	- T1 = Soaking the seeds in plain tap water for 72 h - T2 = Scarification with a hammer	
Time (Day)		
Block		

After sowing, the pots were watered twice a day during cool hours (in the morning and the evening), using the watering can. Regular maintenance (weeding) of the site was carried out. Seeds’ germination was monitored every day for 28 days (Boubacar et al., 2018; Kouame et al., 2012). The seed was considered germinated when the emergence of cotyledons was observed.

The basal diameter (measured using a caliper), the total height (measured with a ruler of 150 cm), and the total number of leaves and ramifications of each seedling were recorded every day-7 (a week) from the day-28 after sowing for three (03) months (September 2020 to January 2021 and from December 2020 to April 2021 respectively for *R. heudelotii* and *B. aegyptiaca* due to the availability period of the seeds of each species).

### 2.4 Data analysis

*Seed mass variability:* Phytodistricts were compared using analysis of variance (ANOVA) followed by a Student-Newman and Keuls tests after verification of assumptions (normality and homoscedasticity). The quantiles (1/3 and 2/3) of the mass of seeds were calculated, and the seeds were grouped into three different mass classes (P1, P2, and P3) for each species.

*Germination rate:* Data were processed using a survival model. To define the survival rate, two variables (“time” and “seeds vital status at the end of the study”) were considered. The time indicated the duration of the seed germination and the duration of observation for non-germinated seeds (right censorship). The second variable indicated the seeds’ vital status at the end of the study (1 = germinated, and 0 = non germinated). The predictor variables which were assumed to influence the germination ability of *B. aegyptiaca* and *R. heudelotii* seeds in the model are phytodistrict, seed mass class (P), and pre-treatment (T). To better appreciate the influence of factors (Table 1) on the germination rate, the analysis of the germination time was used with the non-parametric Kaplan-Meier model (McNair et al., 2012) using “survival” (Therneau 2021) and “survminer” (Kassambara et al., 2021) packages. This model is appropriate in the context of this study which aims to compare the groups of seeds and avoid any dependence of parametric assumptions on the shape of the hazard or survival, without requiring that the time intervals be regular (McNair et al., 2012).

*Initial growth of seedlings:* In this model, the factor “block” was considered to be random, whereas all other factors (“phytodistrict”, “pre-treatment”, and “seed mass class” were considered as fixed (Table 1). Linear and generalized linear mixed-effects models for longitudinal data with a normal distribution of errors were implemented on growth data using “nlme” (Pinheiro et al., 2021) and “MASS” (Venables and Ripley 2002) packages. In these models, provenance (phytodistricts), seed mass class, and pre-treatment were considered as fixed factors and the block as a random factor. The existence of the effect of block and time in the data was tested using the intra-class correlation coefficient (ICC) determined from the empty models (Singer and Willett 2003). To account for temporal autocorrelation, different structures were tested and the one that best fits the data was selected. This selection was based on the Akaike information criterion (AIC) values (low values are preferred, indicating a better fit) using the package “bbmle” (Bolker and Team 2020). The fitted means and standard errors were extracted for each variable and multiple comparisons among them were estimated using “emmeans” package (Lenth 2021). Outputs were used to draw figures using “ggplot2” package (Wickham 2016) in R-4.0.5 software (R Core Team 2021).

## 3 Results

### 3.1 Variability of seeds’ mass of *B. aegyptiaca* and *R. heudelotii* in the study zones

A significant variation was observed for the seed mass for both species (Pr < 2.2e-16) among the sample phytodistricts. The seed mass was higher for seed originated from Atacora chain (3.12 ± 0.77 g) but lower in Mekrou-Pendjari (2.09 ± 0.55 g). For *B. aegyptiaca* and that of *R. heudelotii* was higher in the Plateau phytodistrict (1.89 ± 0.40 g) but lower in the phytodistrict of Pobe (1.14 ± 0.48 g) and South Borgou (1.13 ± 0.23 g). The three seed mass classes were [P1 (P ˂ 2 g and P ˂ 0.75 g), P2 (2 g ≥ P ˂ 3 g and 0.75 g ≥ P ˂ 1.50 g), P3 (P ≥ 3 g and P ≥ 1.50 g)] respectively for *B. aegyptiaca* and *R. heudelotii* (Table 2).

**Table 2:** Minimum (Min), maximum (Max), means and standard errors (Mean ± s.e), Probability of significance (Pr), and seed mass class (P) of *B. aegyptiaca* and *R. heudelotii* in the study zones. Values with different letters are significantly different (apha = 5%, Student Newman and Keuls tests).

Phytodistrict	Min	Max	Mean ± s.e	Pr	Seed mass class (P)
*B. aegyptiaca*					
Atacora chain	1.01	5.56	3.12 ± 0.77^a^	<2.2e-16	P1 = P ˂ 2 g
Mekrou-Pendjari	0.72	3.71	2.09 ± 0.55^c^		P2 = 2 g ≥ P ˂ 3 g
North Borgou	0.79	4.34	2.26 ± 0.74^b^		P3 = P ≥ 3 g
*R. heudelotii*					
Plateau	0.49	2.84	1.89 ± 0.40^a^	<2.2e-16	P1 = P ˂ 0.75 g
Pobe	0.46	1.81	1.14 ± 0.48^b^		P2 = 0.75 g ≥ P ˂ 1.50 g
South Borgou	0.41	2.28	1.13 ± 0.23^b^		P3 = P ≥ 1.50 g

### 3.2 Germination capacity of *B. aegyptiaca* and *R. heudelotii* seeds

**Figure 2:**
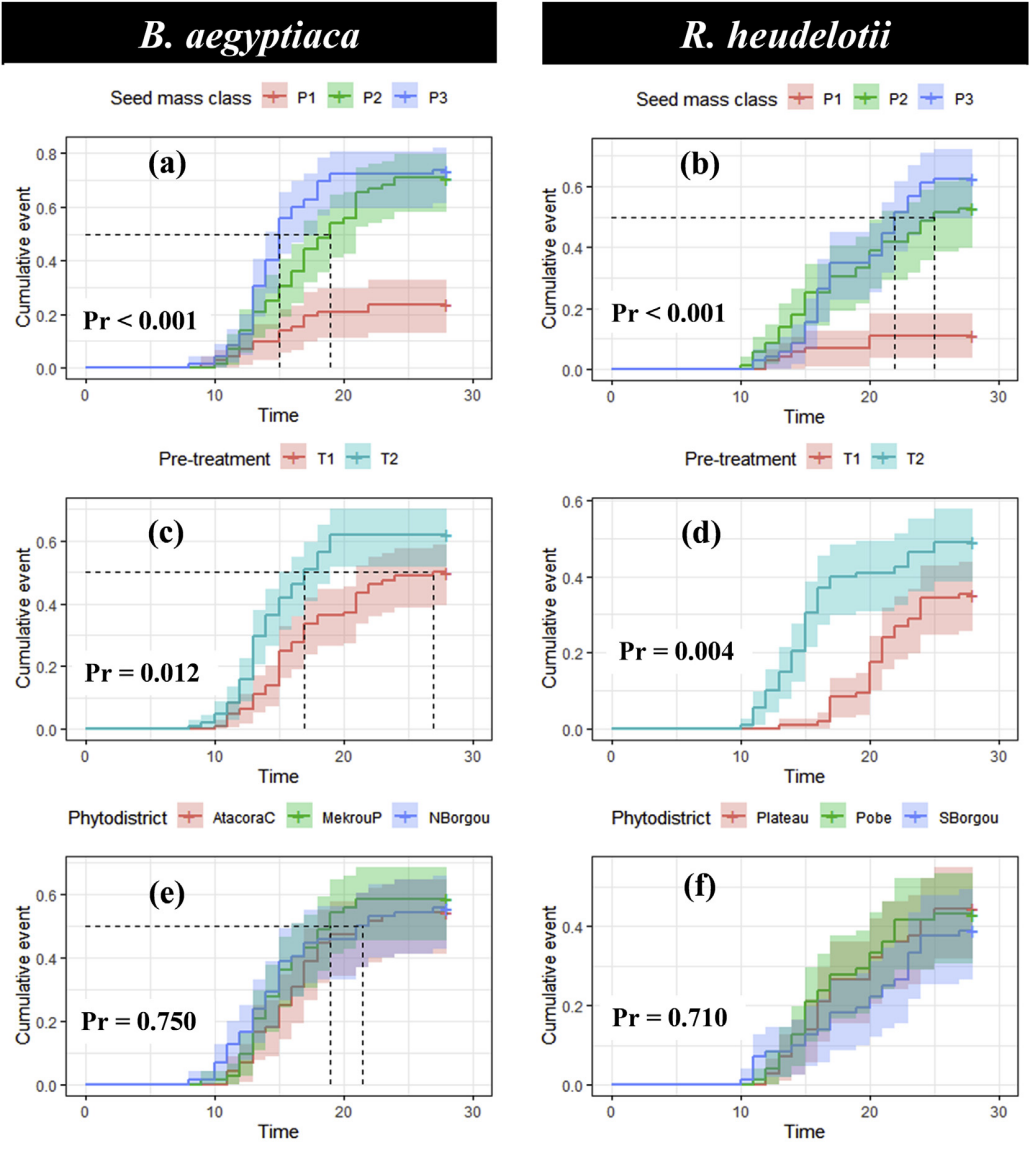
**Evolution trend of germination probability (cumulative event) of *B. aegyptiaca* and *R. heudelotii* seeds according to the seed mass class (a,b), pre-treatment (c,d), and phytodistrict respectively (e,f).** Germination rate = cumulative event value × 100. Seed mass class = P1 (P ˂ 2 g and P ˂ 0.75 g), P2 (2 g ≥ P ˂ 3 g and 0.75 g ≥ P ˂ 1.50 g) and P3 (P ≥ 3 g and P ≥ 1.50 g) respectively for *B. aegyptiaca* and *R. heudelotii,* Pre-treatment technique = T1 (soaking the seeds in plain tap water for 72 h) and T2 (scarification with a hammer), Time = day.

Results showed that the seed mass and pre-treatment have a significant effect on the germination capacity of seeds of *B. aegyptiaca* and *R. heudelotii* (Pr < 0.05; Table 3 & Figure 2).

**Table 3:** Effect of factors on the germination probability of *B. aegyptiaca* and *R. heudelotii* seeds.

Source	βi	exp (βi)	s.e (βi)	z	Pr (>|z|)
*B. aegyptiaca*					
Phytodistrict (*Ref* = Atacora chain)					
Mekrou-Pendjari	0.18	1.19	0.22	0.81	0.415ns
North Borgou	0.19	1.21	0.22	0.86	0.386ns
Seed mass class (*Ref* = P1)					
P2	1.42	4.15	0.28	5.05	4.2e-07∗
P3	1.71	5.54	0.28	6.06	1.2e-09∗
Pre-treatment (*Ref* = T1)					
T2	0.54	1.73	0.18	2.97	0.003∗
Block (Variance)				0.01	
*R. heudelotii*					
Phytodistrict (*Ref* = Plateau)					
Pobe	-0.00	0.99	0.25	-0.00	0.993ns
South Borgou	-0.21	0.81	0.25	-0.81	0.417ns
Seed mass class (*Ref* = P1)					
P2	1.92	6.82	0.39	4.92	8.6e-07∗
P3	2.10	8.20	0.38	5.45	4.7e-08∗
Pre-treatment (*Ref* = T1)					
T2	0.74	2.09	0.21	3.46	0.001∗
Block (Variance)				0.07	

Seed mass class = P1 (P ˂ 2 g and P ˂ 0.75 g), P2 (2 g ≥ P ˂ 3 g and 0.75 g ≥ P ˂ 1.50 g) and P3 (P ≥ 3 g and P ≥ 1.50 g) respectively for *B. aegyptiaca* and *R. heudelotii,* Pre-treatment technique = T1 (soaking the seeds in plain tap water for 72 h) and T2 (scarification with a hammer), βi **=** Regression coefficient, exp = Exponentiate = Hazard ratio (HR), s.e = standard error, z = z statistic, Pr(>|z|) = Probability of significance, ∗ = Significant at 5%, ns = Not significant.

For *B. aegyptiaca,* the heaviest seeds [P3 (P ≥ 3 g)] provided the highest germination rate (73.60 ± 5.19%) and the lowest germination rate (23.60 ± 5.01%) with the fewer heavy seeds [(P1 (P ˂ 2 g)] (Figure 2a). In general, for the three mass classes, the germination rate increased continuously over time for classes P3 and P2 [(2 g ≥ P ˂ 3 g), while for class P1, the germination rate was constant from day-22 (Figure 2a). The seeds scarification with a hammer (T2) gave the first emergence at day-8 and exhibited the highest seed germination rate (62.00 ± 4.67%) compared to pre-treatment T1 which gave a germination rate of 50.00 ± 4.81% with the first emergence at the day-10 (Figure 2c).

As for *B. aegyptiaca*, the trends were similar for *R. heudelotii*. Accordingly, the highest germination rate (62.50 ± 5.71%) was found with the heaviest seeds [P3 (P ≥ 1.50 g)] and the lowest germination rate (11.10 ± 3.70%) with the fewer heavy seeds [P1 (P ˂ 0.75 g)] (Figure 2b). The germination rate increased continuously over time for classes P3 and P2 (0.75 g ≥ P ˂ 1.50 g), while for class P1, the germination rate was constant from day-20 (Figure 2b). The seeds scarification with a hammer (T2) gave the first emergence at day-10 and exhibited the highest seed germination rate (49.10 ± 4.81%) compared to pre-treatment T1 which gave a germination rate of 35.20 ± 4.59% with the first emergence at the day-13 (Figure 2d).

In short, the results showed that whatever the phytodistrict, the seeds of *B. aegyptiaca* and *R. heudelotii* germinated at relatively constant rates (Pr > 0.05; Table 3; Figure 2e, f). The block variance is negligible indicating the weak variation due to the blocks (Table 3).

### 3.3 Initial growth of *B. aegyptiaca* seedlings in the nursery

**Figure 3:**
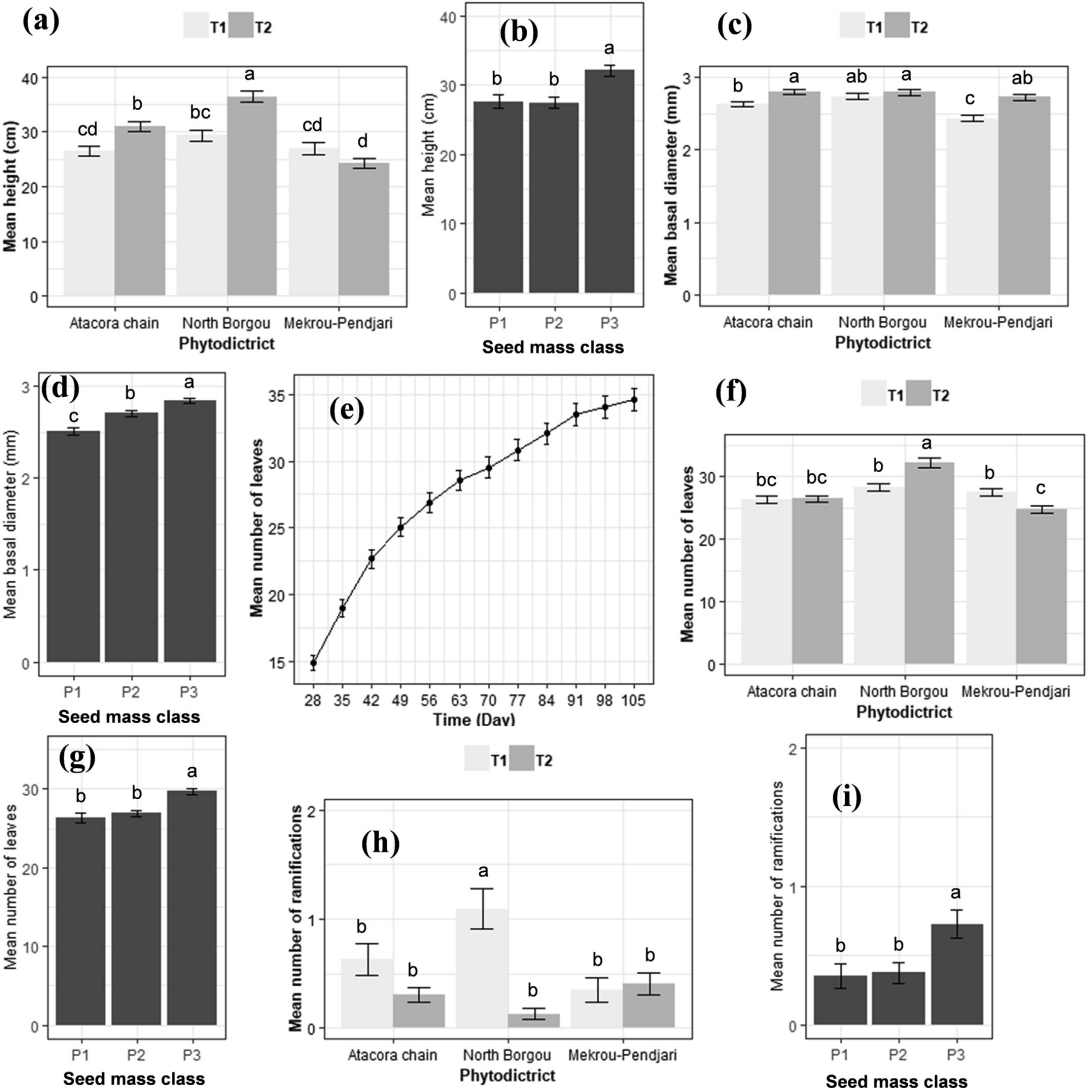
**Evolution trend of the height (a,b), basal diameter (c,d), number of leaves (e,f,g), and ramifications (h,i) of *B. aegyptiaca* seedlings at the end of experiment (day-105).** Seed mass class = P1 (P ˂ 2 g and P ˂ 0.75 g), P2 (2 g ≥ P ˂ 3 g and 0.75 g ≥ P ˂ 1.50 g) and P3 (P ≥ 3 g and P ≥ 1.50 g) respectively for *B. aegyptiaca* and *R. heudelotii,* Pre-treatment technique = T1 (soaking the seeds in plain tap water for 72 h) and T2 (scarification with a hammer).

A significant effect of phytodistrict (provenance) and seed mass was noted on the total height, basal diameter number of leaves, and ramifications of *B. aegyptiaca* seedlings. In addition, the total height varied significantly among the pre-treatment techniques (Pr < 0.05). In addition, the interactions between the phytodistrict, seed mass, and pre-treatment were also significant showing that differences among phytodistricts were not similar across seed mass and pre-treatments. A significant effect of the time was noted on the number of leaves (Pr < 0.05). The block variance is negligible indicating a weak variation due to the blocks (Table 4). Accordingly, the seeds from North Borgou phytodistrict scarified with a hammer (T2) and the heaviest seeds [P3 (P ≥ 3 g)] showed the highest total height (36.43 ± 1.03 cm) and the highest basal diameter (2.84 ± 0.03 mm), the greatest number of leaves (32) and ramifications (1) (Figure 3). While, the lowest values of total height (24.34 ± 0.91 cm), basal diameter (2.43 ± 0.04 mm), number of leaves (25), and ramifications (0) were shown by the seeds from the phytodistrict of Mekrou-Pendjari and the fewer heavy seeds [P1 (P ˂ 2 g)]. On day-28 after sowing, the number of leaves was 15 and was in continuous growth until the end of experiment 35 (day-105) (Figure 3).

**Table 4:** Growth dynamic in height, basal diameter, and number of leaves and ramifications of *B. aegyptiaca* seedlings.

Source	df	Height		Basal diameter		Number of leaves		Number of ramifications	
		F	Pr	F	Pr	Chisq	Pr	Chisq	Pr
Time	11	-	-	-	-	778.17	<2e-16∗	-	-
Phytodistrict	2	32.51	<0.001∗	4.50	0.011∗	22.72	1e-05∗	15.24	0.009∗
Seed mass class (P)	2	46.27	<0.001∗	66.89	<0.001∗	35.74	1e-08∗	16.77	0.004∗
Pre-treatment (T)	1	18.03	<0.001∗	13.31	0.053ns	0.62	0.430ns	14.83	0.080ns
Phytodistrict × P	4	26.08	<0.001∗	23.94	<0.001∗	102.13	<2e-16∗	11.10	0.025∗
Phytodistrict × T	2	29.96	<0.001∗	16.22	<0.001∗	31.62	1e-07∗	16.46	0.000∗
P × T	2	13.27	<0.001∗	32.04	<0.001∗	16.99	0.000∗	**-**	**-**
Block (Variance)		0.09		0.03		0.00		0.01	

df = degree of freedom, P = Seed mass class, T = Pre-treatment technique, df = degree of freedom, F=Fisher statistic, Chisq = Chi square statistic, Pr=Probability of significance, ∗ = Significant at 5%, ns = Not significant.

### 3.4 Initial growth of *R. heudelotii* seedlings in the nursery

**Figure 4:**
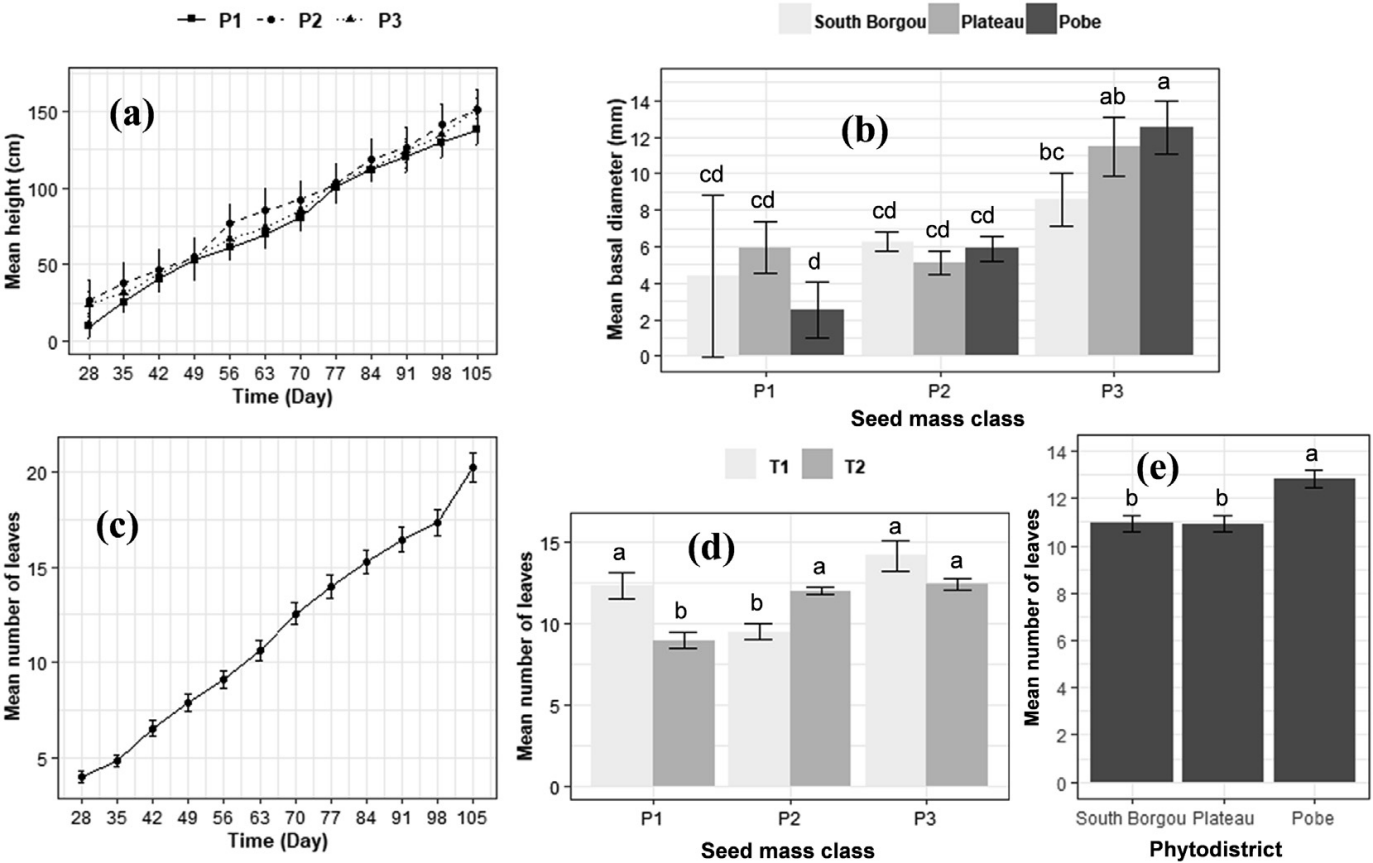
**Evolution trend of the height (a), basal diameter (b), and number of leaves (c,d,e) of *R. heudelotii* seedlings at the end of the experiment (day-105).** Seed mass class = P1 (P ˂ 2 g and P ˂ 0.75 g), P2 (2 g ≥ P ˂ 3 g and 0.75 g ≥ P ˂ 1.50 g) and P3 (P ≥ 3 g and P ≥ 1.50 g) respectively for *B. aegyptiaca* and *R. heudelotii,* Pre-treatment technique = T1 (soaking the seeds in plain tap water for 72 h) and T2 (scarification with a hammer).

Results showed that the variation on the total height of *R. heudelotii* seedlings depends only on the interaction between time and seed mass; thus, indicating that differences among times were not similar across seed masses (Pr < 0.05). A significant effect of the phytodistrict and seed mass and their interaction were noted on the basal diameter (Pr < 0.05). The number of leaves was affected by time, phytodistrict, seed mass, and the interaction between phytodistrict and pre-treatment (Pr < 0.05; Table 5). In particular, the heaviest seeds [ P2 (0.75 g ≥ P ˂ 1.50 g) and P3 (P ≥ 1.50 g)] showed the highest total height from the day-28 after sowing (26.73 ± 13.56 cm and 24.23 ± 7.69 cm respectively) until the end of the experiment (day-105) (150.95 ± 13.26 cm and 151.97 ± 6.37 cm respectively) and the lowest (from 9.81 ± 8.13 cm at the day-28 to 138.17 ± 9.79 cm at the day-105) with the fewer heavy seeds [P1 (P ˂ 0.75 g) (Figure 4a). In addition, the heaviest seeds (P3) from the phytodistrict of Pobe showed the highest basal diameter (12.53 ± 1.47 mm) and the lowest basal diameter (2.54 ± 1.53 mm) with its fewer heavy seeds (P1) (Figure 4b). On day-28 after sowing, the number of leaves was 04 and reached its mean of 20 at day-105 (Figure 4c). The heaviest seeds (P3) from the phytodistrict of Pobe showed also the greatest number of leaves (14), while the fewer heavy seeds (P1) from the phytodistricts of Plateau and South Borgou and the fewer heavy seeds (P1) showed the lowest number of leaves (10) (Figure 4d, e). Moreover, there was not a significant effect of seed pre-treatments (T1 and T2) on the initial growth of *R. heudelotii* seedlings, with almost no ramifications during the trial period (just a single ramified seedling for the whole).

**Table 5:** Growth dynamic in height, basal diameter, and number of leaves of *R. heudelotii* seedlings.

Source	df	Height		Basal diameter		Number of leaves	
		F	Pr	F	Pr	Chisq	Pr
Time	11	0.99	0.912ns	3.84	0.999ns	1080.24	<2.2e-16∗
Phytodistrict	2	0.57	0.563ns	6.11	0.002∗	26.12	2.1e-06∗
Seed mass class (P)	2	0.88	0.413ns	39.33	<0.001∗	28.31	7.1e-07∗
Pre-treatment (T)	1	0.26	0.607ns	2.59	0.107ns	0.06	0.805ns
Time × Phytodistrict	22	0.95	0.521ns	0.43	0.988ns	-	-
Time × P	22	2.14	0.002∗	0.72	0.812ns	-	-
Phytodistrict × P	4	0.43	0.786ns	6.62	<0.001∗	-	-
P × T	2	2.06	0.128ns	6.34	0.052ns	40.15	1.0e-09∗
Block (Variance)		0.03		0.04		0.00	

df = degree of freedom, P = Seed mass class, T = Pre-treatment technique, df = degree of freedom, F=Fisher statistic, Chisq = Chi square statistic, Pr=Probability of significance, ∗ = Significant at 5%, ns = Not significant.

## 4 Discussion

This study revealed that the seeds of *B. aegyptiaca* were heavier in the phytodistrict of Atacora chain but lighter in the phytodistrict of Mekrou-Pendjari. As for *R. heudelotii*, its seeds were heavier in the phytodistrict of Plateau but lighter in the phytodistricts of Pobe and South Borgou. In addition, the trend of the seed mass recorded was similar across phytodistricts. This revealed the representativeness of the seed masses and made it possible to group them into three classes. This observation suggested that environmental factors (particularly rainfall) play an important role in determining the seed characteristics as well as the probable influence of genetic factors. Several studies pointed out that plants growing in areas with high rainfall tend to develop higher seed masses (Hounsou-Dindin et al., 2016; Padonou et al., 2013). Investigations in a controlled environment (provenance test) were, therefore, necessary to confirm the heritability of the characters observed. Whenever these characters turn out to be highly hereditary, they could be used in the definition of descriptors to characterize the "varieties" or ecotypes that would come to be identified.

The seeds of *B. aegyptiaca* and *R. heudelotii* trees most often take longer to germinate (because of their maturity which comes only at the end of the rainy season), compared to the ones of grasses and other cultivated plants (which mature during the rainy season, e.g., *Mangifera indica*) which germinate quite quickly, due to various intrinsic factors such as seed coat or physiological dormancy. Our findings were that the scarification of the seeds with a hammer (T2) gave the first emergence for relatively short times at the day-8 and day-10 with the highest seed germination rates (62.00 ± 4.67% and 49.10 ± 4.81%) compared to pre-treatment (T1 = Soaking the seeds in plain tap water for 72 h) which gave germination rates of 50.00 ± 4.81% and 35.20 ± 4.59% with its first emergence at day-10 and day-13 respectively for *B. aegyptiaca* and *R. heudelotii*. The findings were supported by several other studies which showed that germination of species is subject to various integumentary dormancy hinders the germination seeds and aligned with previous studies reported by Boubacar et al. (2018); Kouyaté et al. (2015); Djeugap et al. (2013); Kouame et al. (2012) and Moussa et al. (2020). This confirms that the seed dormancy of these species is physical and scarification of the seed coat is necessary to promote seed imbibition and germination. In addition, the results revealed highly significant differences (Pr < 0.05) between the germination rate of the seed masses, indicating that the heaviest seeds provided the highest germination rates (73.60 ± 5.19% and 62.50 ± 5.71%) and the lowest germination rates (23.60 ± 5.01% and 11.10 ± 3.70%) were obtained with the lighter seeds [(P1 (P ˂ 2 g) and P1 (P ˂ 0.75 g)] respectively for *B. aegyptiaca* and *R. heudelotii.* These results confirm that the large-sized seeds germinated faster than smaller seeds (Das et al., 2016; Padonou et al., 2013) due to the quantity of the stored reserve of substances. It is important to pinpoint that germination rates do not vary significantly among phytodistricts; thus, revealing that seeds collected from one area or another of the country gave satisfactory results. Nevertheless, it is necessary to underline the advantages and disadvantages associated with each pre-treatment technique. For example, the scarification with a hammer (T2) showed a high germination rate but requires more time, manpower (if the seeds are a lot), adequate material (the mass of the hammer and the quality/solidity of the support), and rigorous technical know-how to avoid crushing the seeds. Soaking the seeds in plain tap water for 72 h (T1) showed relatively low rates but was easy and not demanding. Thus, we can recommend T2 for small-scale production (less than 100 seeds) and for T1 for large-scale production. The exclusion of chemical techniques is primarily due to the lack of a device to neutralize debris or chemical deposits, both in humans who handle them but also in production. This constitutes a great risk for public health (from producer to consumer) of which the most cited dangerous diseases are: cancer, mutations, or reproductive problems (Samb and Schiffers 2003). The seed viability test is important to effectively guarantee good germination rates and the preferred use of freshly harvested seeds. Indeed, similar trends were observed at the level of the initial growth of *B. aegyptiaca* and *R. heudelotii* seedlings. The seeds scarified with a hammer (T2) and the heaviest seeds (P3) showed the highest total height and indicated the importance of the seed size in the plant growth. These may be due to the accumulation of reserve in the albumen or directly in the cotyledons (Das et al., 2016). All seeds contain reserves that are used by the embryo at emergence stage (i.e., before the photosynthetic apparatus is differentiated) and therefore, that the young seedling becomes autotrophic. When a seed germinates, it has only two days (48 h) before having exhausted its reserves to transform into a seedling capable of photosynthesis (FAO 1992). As the relative speed of use of reserves varies according to the mass of the original seed (Pommel and Bouchard 1990), seedlings from large seeds mobilize in a given time a much greater quantity of reserves and therefore grow faster than those from small seeds. Seed vigor is an important requirement for good germination and seedlings’ growth. In the case of a small seed, the quantity of the reserves is low and render difficult the germination. However, these constitute a safety margin: seedlings from small seeds will show greater fragility vis-à-vis environmental conditions. Thus, knowledge of the mass of the original seed, which determines the capacity of the seedling to develop and its resistance to environmental conditions is, therefore, an essential element in characterizing the performance of a seedling.

The growth of vegetative organs is among processes that allow plants to grow and to optimize the surfaces of exchanges with the external environment, characteristics of their strategy of exploitation of resources. Variables measured in seedlings from different provenances revealed significant differences between provenances. Particularly, *B. aegyptiaca* seeds from North Borgou phytodistrict and those of *R. heudelotii* from the phytodistrict of Pobe were the ones that showed a high growth performance. The observed variabilities across the different phytodistricts may be a result of combined actions of geographic isolation and gene mutation (Diallo et al., 2010). The present results aligned with previous studies that reported the rapid seedling growth is more related to genetic factors of parent trees than to soil conditions and environmental factors (Weber et al., 2015) and may serve as a selection trait for genetically superior progeny. Variations in the pre-treatments would be due to the shorter germination times compared to each other, thus offering the advantage to the seedlings from the seeds scarified with a hammer (T2) to grow faster. This solves the problem of species domestication with more appropriate techniques.

## 5 Conclusion

This study revealed the importance of choosing the heaviest seeds to facilitate and guarantee a high germination rate of seeds and seedling growth of *Balanites aegyptiaca* (seed mass ≥3 g) and *Ricinodendron heudelotii* (seed mass ≥1.50 g)*.* In addition, these seeds need to be pre-treated by scarification with a hammer or soaking in plain tap water for 72 h before sowing. Nevertheless, the scarification of the seeds with a hammer provides a high plant production performance for these species. Absolute seedling growth is proportional to the seed size, at least at the establishment stage. This study is of great contribution to the development of income-generating activities for local communities and soil restoration through agroforestry systems diversification in the context of climate change. Future research should assess these species’ genetic diversity, biochemical characteristics, and value chains and identify the proper propagation techniques.

## Declarations

### Author contribution statement

Guillaume Hounsou-Dindin; Rodrigue Idohou: Conceived and designed the experiments; Performed the experiments; Analyzed and interpreted the data; Contributed reagents, materials, analysis tools or data; Wrote the paper.

Marcel T. Donou Hounsode; Romain Glèlè Kakaï: Conceived and designed the experiments; Analyzed and interpreted the data; Contributed reagents, materials, analysis tools or data; Wrote the paper.

Aristide Cossi Adomou; Achille Ephrem Assogbadjo: Conceived and designed the experiments; Contributed reagents, materials, analysis tools or data; Wrote the paper.

### Funding statement

This work was supported by the University of Abomey-Calavi (Project ‘WILD-OIL/2018/PFCR III/UAC’) for a National PhD fellowship.

### Data availability statement

Data will be made available on request.

### Declaration of interests statement

The authors declare no conflict of interest.

### Additional information

No additional information is available for this paper.
